# Adaptive Fusion Based Method for Imbalanced Data Classification

**DOI:** 10.3389/fnbot.2022.827913

**Published:** 2022-02-28

**Authors:** Zefeng Liang, Huan Wang, Kaixiang Yang, Yifan Shi

**Affiliations:** ^1^School of Computer Science and Engineering, South China University of Technology, Guangzhou, China; ^2^Guangdong Institute of Scientific and Technical Information, Guangzhou, China; ^3^State Key Laboratory of Industrial Control Technology, Zhejiang University, Hangzhou, China; ^4^College of Engineering, Huaqiao University, Quanzhou, China

**Keywords:** imbalance learning, metric learning, information fusion, classification, ensemble learning

## Abstract

The imbalance problem is widespread in real-world applications. When training a classifier on the imbalance datasets, the classifier is hard to learn an appropriate decision boundary, which causes unsatisfying classification performance. To deal with the imbalance problem, various ensemble algorithms are proposed. However, conventional ensemble algorithms do not consider exploring an effective feature space to further improve the performance. In addition, they treat the base classifiers equally and ignore the different contributions of each base classifier to the ensemble result. In order to address these problems, we propose a novel ensemble algorithm that combines effective data transformation and an adaptive weighted voting scheme. First, we utilize modified metric learning to obtain an effective feature space based on imbalanced data. Next, the base classifiers are assigned different weights adaptively. The experiments on multiple imbalanced datasets, including images and biomedical datasets verify the superiority of our proposed ensemble algorithm.

## 1. Introduction

Many applications face imbalance problems (Farrand et al., 2020; Khushi et al., 2021; Zhang et al., 2021). The imbalance problem is caused by the difference in the number of samples in each class. When the classifiers are trained on imbalanced datasets, the classifiers tend to favor the majority class and predict more samples to be the majority class. Therefore, the minority class samples can not be correctly classified, which is called the imbalance problem. The imbalance problem is widespread in the applications, so more and more researchers focus on dealing with the imbalance problem.

To solve the imbalance problem, researchers have proposed various methods from different perspectives. Cost-sensitive method (Elkan, 2001) is a typical one. The cost-sensitive method assigns different classification losses to each class. The minority class has a higher classification loss than the majority class, such that the classifiers pay more attention to the minority class and get a correct result. Resampling is another typical method. Resampling methods remove or synthesize samples from the original data to balance the number of samples in each class, including undersampling, oversampling, and hybrid sampling. Undersampling (He and Garcia, 2009) method removes the majority class samples by some informed rules. Undersampling can produce a more clear decision boundary while the information of the excluded samples is lost.

On the other hand, the oversampling method proposes to generate the synthesis of minority class samples until the data is balanced. The synthesis samples may lie in the overlapped area and make the distribution worsen. To overcome the disadvantages, the hybrid sampling is investigated. As a two-stage strategy, hybrid sampling method combines undersampling and oversampling (I., 1976; Han et al., 2005). Other researchers combine the clustering method with oversampling (Barua et al., 2011).

Ensemble learning is widely used in solving the imbalance problem. Ensemble learning trains different classifiers and gets the result by integrated voting, which contains boosting and bagging (Ho, 1998; Skurichina and Duin, 2002; Chawla et al., 2003; Wang and Yao, 2009; Chen et al., 2010). Ensemble learning plays an essential role in the imbalance classification tasks (Bi and Zhang, 2018).

Nevertheless, the traditional imbalance algorithms have the following problems. Most algorithms do not consider mapping the imbalance data to another feature space for better classification performance. In addition, the importance of base classifiers is different, so it is inappropriate to treat the voting weight of base classifiers equally.

Metric learning is a hot topic in machine learning, which has been utilized in practical applications (Cao et al., 2019; Bai et al., 2021). Metric learning learns a feature space that is more effective than the original space. Euclidean distance is a common measure. However, Euclidean distance can not reflect the relationship correctly in the overlapped area. Consequently, some researchers capture the distance between samples by finding a transformation that can increase the distance between dissimilar samples and reduce the distance of similar samples (Köstinger et al., 2012). When training on the imbalance datasets, metric learning also suffers from imbalance problems (Gautheron et al., 2019). It needs to be modified before training on the imbalance datasets.

In this article, we propose an ensemble learning framework that combines metric learning and resampling. The metric learning is employed by building a feature space from the imbalanced dataset. The classifier is trained on the balanced datasets after oversampling on the feature space to reduce the impact of imbalance. Finally, the classifier is integrated by adaptive weighted voting.

The contributions of the article are as follows:

1) An imbalanced version of the large margin nearest neighbor (LMNN) algorithm is proposed to alleviate the influence of imbalanced data distribution and learn a robust feature space.

2) An GA-based weighting scheme is designed to adaptively optimize the importance of different classifiers.

3) Extensive experiments are conducted on various imbalanced datasets to verify the effectiveness of the proposed approach.

The main framework is as follows: Section 2 introduces the related work about resampling, ensemble learning, and metric learning; Section 3 discusses the proposed ensemble framework in detail; Section 4 shows the experiments about our proposed methods and discusses the result of the experiments. Section 5 draws the conclusion and future study.

## 2. Related Work

The resampling method contains undersampling and oversampling. To get a balanced dataset, undersampling methods remove the majority of samples randomly or by informed rules. Tomek link (Batista et al., 2004) removes samples that are of a different class from the neighbor. Undersampling can reduce the imbalance problem, while it may suffer from information loss. When the number of samples in each class is quite different, most of the majority of class samples are removed. Hence, the information of the majority of class is lost severely. On the other hand, oversampling proposes to generate synthesis minority class samples to balance the dataset. SMOTE (Chawla et al., 2002) propose to generate samples by interpolating between a given sample and its neighbors. The synthesis sample $x_{i}^{*}$ is generated as follows:


(1) $$\begin{array}{l} x_{i}^{*}=x_{i}+\left(x_{n}-x_{i}\right)*r \end{array}$$

In which *x*_*n*_ is the neighbor of sample *x*_*i*_ and *r* is a random value between [0, 1]. Adaptive Synthetic sampling approach (ADASYN) (He et al., 2008) makes different samples generate different numbers of synthetic samples. Some methods combine oversampling with clustering to overcome the problem that synthesized samples are located in the overlapped area. Majority Weighted Minority Oversampling Technique (MWMOTE) (Barua et al., 2014) generates minority samples within the cluster. Additionally, geometric-SMOTE (Douzas and Bacao, 2019) proposes a universal method that can be used in most oversampling methods. Mahalanobis Distance-based Over-sampling technique (MDO) (Abdi and Hashemi, 2016) and its variant (Yang et al., 2018) propose to generate samples in the principal component space.

Euclidean distance is a traditional measure to reflect the similarity between samples. However, dissimilar samples may be closer to the similar samples in the overlapped area, which is inefficient to apply Euclidean distance. Metric learning learns a feature space that can reflect the relationship between samples more correctly. In the feature space, similar samples are closer while dissimilar samples are separated apart. To achieve this goal, many metric learning algorithms have been proposed. LMNN (Weinberger and Saul, 2009) minimizes the distance between the anchor sample and its neighbors of the same class. At the same time, the anchor sample maintains a margin with neighbors of a different class. Information-theoretic metric learning (ITML) (Davis et al., 2007) makes distribution on the feature space similar to the Gaussian distribution. Some methods utilize metric learning on imbalanced datasets. Imbalance metric learning (IML) (Gautheron et al., 2019) modified LMNN by assigning different weights to sample pairs. Distance Metric by Balancing KL-divergence (DMBK) (Feng et al., 2019) balances the divergence of each class on the feature space. Iterative metric learning (Wang et al., 2018) learns a feature space for the area near each testing data.

Ensemble learning integrates classifiers to improve the robustness and performance of classification results. EasyEnsemble (Liu et al., 2009) trains several classifiers on the subset, which contains part of majority class samples and whole minority class samples. BalanceCascade (Liu et al., 2009) splits the majority class samples as several subsets and trains AdaBoost classifiers based on the subsets. Yang et al. (2021) proposes an ensemble framework based on subspace feature space ensemble and metric learning.

## 3. Proposed Methodology

**Figure 1:**
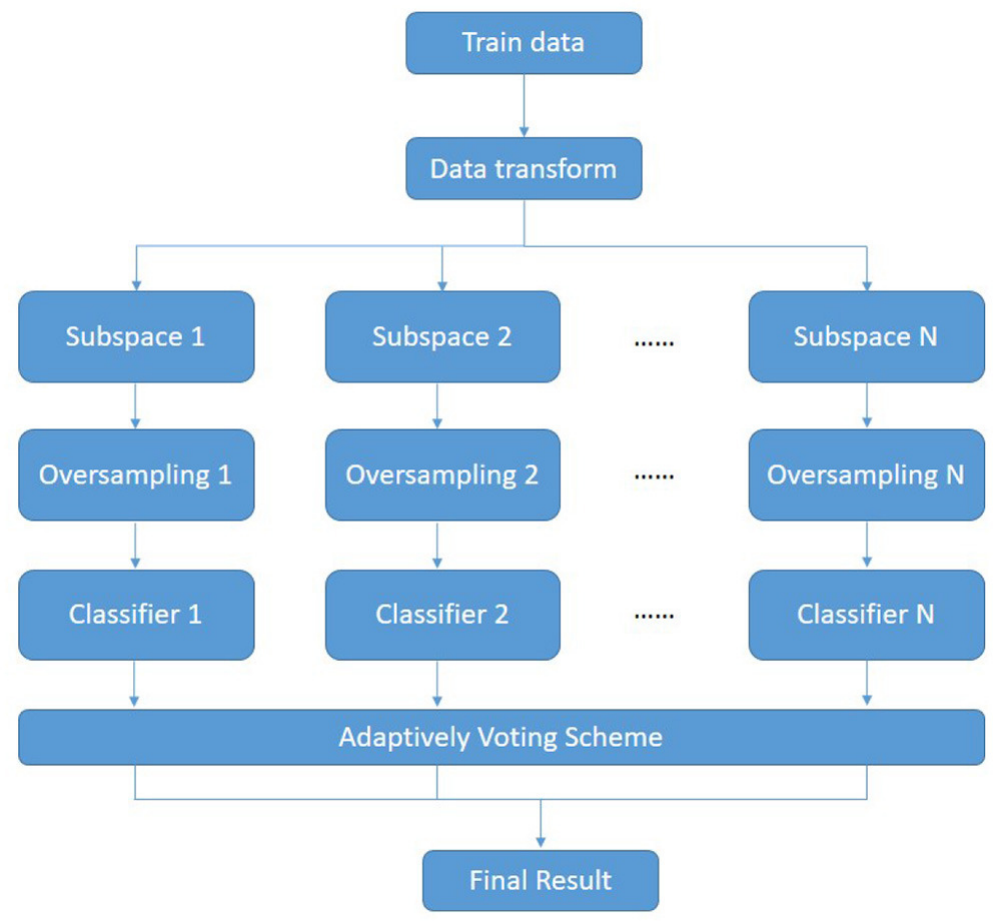
The overall framework of ensemble algorithm.

In this section, we propose an ensemble framework combining metric learning with oversampling. Figure 1 shows the overall framework of our proposed algorithm. First, the metric learning methods based on the imbalance problem(denoted as ImLMNN) are applied for getting a better feature space *L*. The data *X* is transformed by mapping matrix *L* and gets the mapped data X^*^. Then, the feature space *S*_*i*_ is constructed. Next, the oversampling method is employed for getting a balance training dataset $S_{i}^{*}$. Finally, different classifiers are applied in balance datasets and voting for the result. The pseudo-code is shown in the Algorithm 1.

**Algorithm 1:** Imbalance Ensemble Framework.

**Input:** Training set **X** = (*x*_1_, *x*_2_, …, *x*_*n*_)
**Parameter:** Number of subspace *N*
**Procedure:**
1: Obtain the feature space **L** by ImLMNN;
2: Map the data by learned feature space **L** and get the mapped dataset X^*^;
3: **for** *i* in 1,…, *N* **do**
4: Extract part of features by a threshold *M* and get the subspace **S**_*i*_;
5: Apply SMOTE oversampling method on subspace **S**_*i*_ and get the balanced subset ${\text{S}}_{i}^{*}$;
6: Classify on subset ${\text{S}}_{i}^{*}$ and get predict result $y_{i}^{*}$;
7: **end for**
8: Vote for result by adaptive weight **W** = [*w*_1_, *w*_2_, …, *w*_*N*_];
**Output:** The final result $\text{Y}=\left(y_{final}^{1},y_{final}^{2},\ldots ,y_{final}^{n}\right)$.

We aim at finding a feature space that can better describe the sample relationship to improve the performance of classifiers. LMNN transforms data to a latent feature space, in which similar samples are closer while dissimilar samples are separated apart. The loss function of LMNN is as follows:


(2) $$\begin{array}{l} f\left(\text{L}\right)=f_{push}\left(\text{L}\right)+f_{pull}\left(\text{L}\right) \end{array}$$

where


(3) $$f_{pull}(L)=\sum_{i,j}{\left\|L\left(x_{i}-x_{j}\right)\right\|}^{2}$$

(4) $$f_{push}(L)=\sum_{i,j}\sum_{l}\left(1-y_{il}\right){\left[1+{\left\|L\left(x_{i}-x_{j}\right)\right\|}^{2}-{\left\|L\left(x_{i}-x_{l}\right)\right\|}^{2}\right]}_{+}$$

The loss function of LMNN contains *f*_*push*_(**L**) and *f*_*pull*_(**L**). *f*_*pull*_(**L**) reduces the distance between the anchor and its similar neighbors, while *f*_*push*_(**L**) penalizes the distance between dissimilar samples. [*z*]_+_ = max(*z*, 0) is the hinge loss. *y*_*il*_ = 1 when *x*_*i*_ and *x*_*l*_ belong to the same class, otherwise *y*_*il*_ = 0.

However, the LMNN algorithm is inappropriate directly to apply in imbalanced datasets. To solve this problem, we assign different weights to samples. The weight *w*_*i*_ of sample *x*_*i*_ is as follows:


(5) $$\begin{array}{l} w_{i}=\frac{\delta^{i}}{|N_{c}|*d\left(x_{i},\bar{X_{c}}\right)} \end{array}$$

The loss of samples is divided by the number of samples *N*_*c*_ in the corresponding class, such that the impact caused by the imbalance problem is alleviated. To emphasize the samples near decision boundaries, we compute the sum of the density of majority class $\delta_{n}^{i}$ and minority class $\delta_{p}^{i}$ as density δ^*i*^. The density δ^*i*^ is defined as follows:


(6) $$\begin{array}{l} \delta^{i}=\delta_{n}^{i}+\delta_{p}^{i} \end{array}$$

(7) $$\begin{array}{l} \delta_{n}^{i}=\frac{1}{\frac{1}{k}\overset{k}{\underset{j=1}{\sum }}d_{ij}},\;\delta_{p}^{i}=\frac{1}{\frac{1}{h}\overset{h}{\underset{j=1}{\sum }}d_{ij}} \end{array}$$

$\delta_{n}^{i}$ and $\delta_{p}^{i}$ describe the aggregation of samples in neighboring areas about majority class and minority class. *k* and *h* are the number of neighbor samples in calculating $\delta_{n}^{i}$ and $\delta_{p}^{i}$, respectively. When the density $\delta_{c}^{i}$ is large, the samples in class *c* are close to *x*_*i*_. Therefore, a large sum of density δ^*i*^ reflects that sample *x*_*i*_ is close to samples of both majority and minority classes or in the inner of class with high density.

Outliers and noises are also in the border area. To alleviate the influence of outliers and noises, we divide sample weight by $d\left(x_{i},\bar{X_{c}}\right)$ which is the distance between sample *x*_*i*_ and the center of class *c*. The center of class *c* is defined as:


(8) $$\begin{array}{l} \bar{X_{c}}=\underset{\left\{i|y_{i}=c\right\}}{\sum }x_{i}. \end{array}$$

Therefore, the overall objective function of the data transformation algorithm is:


(9) $$\begin{array}{l} f(L)=\sum_{i,j}w_{i}{\left\|L\left(x_{i}-x_{j}\right)\right\|}^{2}\\ \;+\sum_{i,j}\sum_{i}w_{i}\left(1-y_{il}\right){\left[1+{\left\|L\left(x_{i}-x_{j}\right)\right\|}^{2}-{\left\|L\left(x_{i}-x_{l}\right)\right\|}^{2}\right]}_{+} \end{array}$$

**Figure 2:**
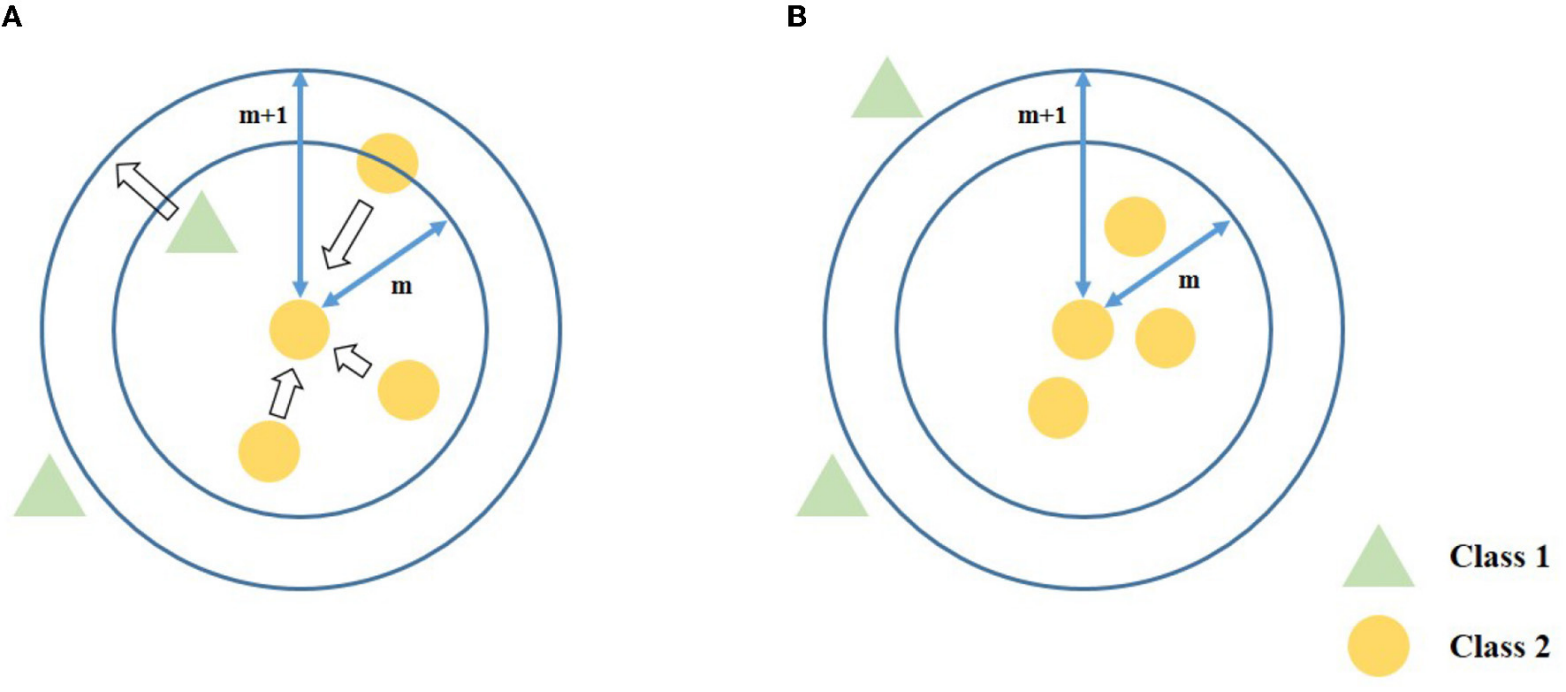
The diagram of proposed data transformation. After the data transformation, the similar neighbors are closer, while the dissimilar neighbor samples are pushed and hold a certain distance to the anchor sample. **(A)** Before data transformation. **(B)** After data transformation.

The diagram of data transformation is shown in Figure 2. Similar to LMNN, Equation (9) contains *f*_*pull*_ which pulls similar samples closer and *f*_*push*_ which push dissimilar samples separate apart. In addition, each sample has a different weight to deal with the imbalance problem.

After the data transformation, we extract *M* features to build the subspace **S**_*i*_:


(10) $$\begin{array}{l} {\text{S}}_{i}=\left[f_{i}^{1},f_{i}^{2},...,f_{i}^{M}\right] \end{array}$$

The feature is extracted *N* times to generate *N* subspace. In the subspace, the dataset is still imbalanced, which affects the classifier's performance. To solve this problem, oversampling is utilized in each feature subspace. Specifically, the subset is ${\text{S}}_{i}^{*}={\text{S}}_{i}\cup {\text{S}y\text{n}}_{i}$. The **Syn**_*i*_ is the synthesis minority data that is generated by SMOTE on feature subspace **S**_*i*_ and helps to form a new balanced subset ${\text{S}}_{i}^{*}$ with the original subspace.

The classifier *c*_*i*_ is trained on the balance subset ${\text{S}}_{i}^{*}$ and votes for obtaining the result. However, the performance of each classifier is different. The contribution of each classifier in voting should be determined by the classification result, rather than being treated equally. Therefore, we utilize the weight assign process to progressively vote. In detail, the GA algorithm is applied to obtain the weight of each classifier adaptively. The detailed description is shown as Algorithm 2.

**Algorithm 2:** Adaptive weight Procedure.

**Input:** Classifier set **C** = (*c*_1_, *c*_2_, …, *c*_*n*_)
**Parameter:** Number of genes *n*, Population size of genes *n*_*p*_, Max iteration *N*
**Initialize:** genes **G** = (*g*_1_, *g*_2_, …, *g*_*n*_)
**Procedure:**
1: **while** not converge **do**
2: Select genes as parent randomly;
3: Do crossover and mutation on parent genes to generate *n*_*child*_ child genes by Eq. (11) and Eq. (12);
4: **for** *i* in 1,…,*n* **do**
5: Calculates the fitness of gene *Fparent*_*i*_;
6: **end for**
7: **for** *j* in 1,…,*n*_*child*_ **do**
8: Calculates the fitness of gene *Fchild*_*j*_;
9: **end for**
10: **if** *Fchild*_*h*_ >*Fparent*_*l*_ **then**
11: replace parent gene *g*_*l*_ by child gene *g*_*h*_;
12: **end if**
13: **Until** *N* **Converge**
14: **end while**
**Output:** The optimal genes ${\text{G}}^{*}=\left(g_{1}^{*},g_{2}^{*},\ldots ,g_{n}^{*}\right)$.

First, the initial genes **G** = (*g*_1_, *g*_2_, …*g*_*n*_) is generated as the weight of the classifier, in which *n* is the number of subspace and *g*_*i*_ is the weight of classifier *i*. Next, the GA algorithm finds the *n* individual and does crossover and mutation. Given two parent genes $g_{i}=\left[p_{i}^{1},p_{i}^{2},...,p_{i}^{S}\right]$ and $g_{j}=\left[p_{j}^{1},p_{j}^{2},...,p_{j}^{S}\right]$ with length *S*, the crossover method exchanges part of features in genes. Suppose the exchange occurs at position α (α ∈ [1, *S*]), then the genes after the exchange are:


(11) $$\begin{array}{c} g_{i}^{*}=\left[p_{i}^{1},p_{i}^{2},...,p_{j}^{\alpha },...,p_{i}^{S}\right]\\ g_{j}^{*}=\left[p_{j}^{1},p_{j}^{2},...,p_{i}^{\alpha },...,p_{j}^{S}\right] \end{array}$$

The mutation may occur in each position of genes. Suppose the mutation happens in position γ (γ ∈ [1, *S*]) of gene *g*_*k*_, then we have:


(12) $$\begin{array}{c} g_{k}^{*}=\left[p_{k}^{1},p_{k}^{2},...,p_{k}^{\gamma },...,p_{k}^{S}\right] \end{array}$$

in which $p_{k}^{\gamma }$ is a random value. After crossover and mutation, the child's genes are generated. We calculate the fitness of parent genes *Fparent*_*l*_ (*l* ∈ [1, *n*]) and child genes *Fchild*_*h*_ (*h* ∈ [1, *n*_*child*_]). The fitness is set as AUC value. Finally, the parent genes are replaced by child genes with higher fitness values. When the iteration is over, the optimal classifier weight ${\text{G}}^{*}=\left(g_{1}^{*},g_{2}^{*},...,g_{n}^{*}\right)$ is obtained.

We can get the final result by weighted voting integration. The result of classifier *c*_*i*_ is denoted as *y*_*i*_. Then, the final result is


(13) $$\begin{array}{l} y_{final}=\underset{N}{\sum }g_{i}^{*}y_{i} \end{array}$$

## 4. Experiment

In this section, we show the experiments about the proposed ensemble framework and compare the algorithm on various datasets from UCI (Dua and Graff, 2017) and KEEL (Alcala-Fdez et al., 2010). Our algorithm is also applied in the Fashion-mnist image dataset. Finally, we analyze the effect of parameters on our proposed algorithm.

### 4.1. Datasets

To evaluate the performance of our algorithm, we choose eight datasets from UCI and KEEL with different attributes, such as imbalance ratio (IR), number of samples, and features. The attributes are shown in Table 1 in detail.

**Table 1:** The attributes of datasets.

	**IR**	**Samples**	**Features**
climate	10.74	540	18
libras_move	14.00	360	90
ecoli2	5.46	90	7
glass_0_1_2_3_vs_4_5_6	3.20	214	9
yeast3	8.10	1484	8
cleveland_0_vs_4	12.31	173	13
winequality_red_4	29.17	1599	11
ecoli1	3.36	336	7

### 4.2. Evaluation Criteria

Accuracy is the typical criterion to evaluate the performance of the algorithm. However, due to the imbalance problem, accuracy is inappropriate for imbalance learning. AUC (Fawcett, 2004) is the area under the receiver operating characteristic curve, which is not sensitive to the imbalance data, and it is widely used in imbalance learning. In the experiments, we use AUC as evaluation criteria.

### 4.3. Comparison With Other Algorithms

To show the superiority, several algorithms and imbalance ensemble frameworks are compared with our algorithm. Specifically, we choose RandomForest, RUSboost, and BalanceBagging as the baseline. In addition, SMOTE algorithm is also chosen. The baseline algorithms are shown as follows:

1. SMOTE: A typical oversampling method. It generates samples by interpolating between samples and their neighbors.
2. RandomForest: An ensemble framework that uses bagging to build subsets for tree classifiers. The number of trees we set is 15.
3. RUSboost: A hybrid method that combines sampling with boosting. The number of iterations is 15.
4. BalanceBagging: A variant of Bagging that is applied sampling in each bootstrap. The number of subspaces we set is 15.

For our proposed algorithm, the number of subspaces is 15, and the ratio of extracted features is 0.7. To show the ablation experiment, we compare the LMNN ensemble, which replaces our proposed data transformation algorithm ImLMNN with the original LMNN algorithm. We choose linear SVM as the base classifier. The algorithms run five times and calculate average AUC as evaluation criteria. The 5-fold cross-validation is also applied. The result of the experiment is shown in Table 2.

**Table 2:** Comparisons between imbalance learning algorithm and our proposed method in terms of AUC.

	**SMOTE**	**RandomForest**	**RUSboost**	**Balance bagging**	**LMNN ensemble**	**ImLMNN ensemble**
climate	0.8785 ± 0.0092	0.5353 ± 0.0188	0.7462 ± 0.0245	0.8496 ± 0.0206	0.8787 ± 0.0162	**0.8796 ± 0.021**
libras_move	0.8741 ± 0.0227	0.8133 ± 0.025	0.8015 ± 0.06	0.8544 ± 0.0153	0.8146 ± 0.0286	**0.8975 ± 0.0144**
ecoli2	0.8671 ± 0.0121	0.813 ± 0.0113	0.8252 ± 0.0504	0.8678 ± 0.0138	0.8537 ± 0.0102	**0.873 ± 0.0086**
glass_0_1_2_3_vs_4_5_6	0.8945 ± 0.0172	0.876 ± 0.0129	0.8441 ± 0.039	0.8804 ± 0.0251	0.827 ± 0.0295	**0.9064 ± 0.0173**
yeast3	0.8278 ± 0.8278	0.7348 ± 0.0219	0.8163 ± 0.0258	0.8256 ± 0.0273	0.8381 ± 0.0163	**0.8395 ± 0.0125**
cleveland_0_vs_4	0.8919 ± 0.0103	0.6367 ± 0.088	0.7547 ± 0.05	0.848 ± 0.0405	0.8871 ± 0.0236	**0.8955 ± 0.0419**
winequality_red_4	0.6667 ± 0.0175	0.5291 ± 0.0093	0.5919 ± 0.0323	0.6515 ± 0.6515	0.6615 ± 0.0111	**0.6776 ± 0.0116**
ecoli1	0.8715 ± 0.0208	0.8461 ± 0.0297	0.7868 ± 0.0464	0.8756 ± 0.0228	0.8786 ± 0.0062	**0.8804 ± 0.0117**
AVERAGE_AUC	0.8465	0.723	0.7708	0.8316	0.8299	**0.8562**

*The bold value means the best result among the compared algorithms*.

From Table 2, we can see that our algorithm has the highest average AUC on given datasets, which is superior to other compared algorithms. Compared with other algorithms, the proposed algorithm has at least a 1% improvement in average AUC. Also, compared with the ensemble algorithm that applied the original LMNN algorithm as data transformation, our proposed method has a near 3% improvement in average AUC. Our method takes data transformation in the imbalanced datasets into account, which is superior to other compared algorithms.

### 4.4. Comparison With Different Algorithms on Image Dataset

**Figure 3:**
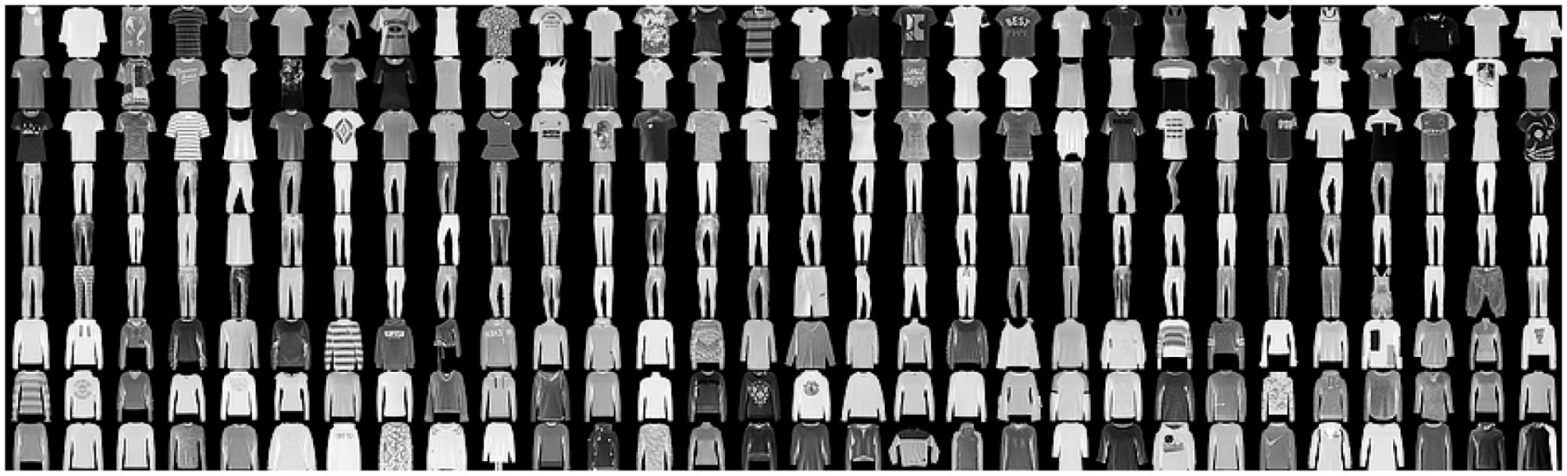
The Fashion-mnist dataset.

Our algorithm is applied in the image dataset and compared with other algorithms. Figure 3 shows part of samples in the Fashion-mnist dataset. Fashion-mnist is a famous image dataset that has 784 features and 60,000 samples. The dataset has 10 classes. To build the imbalanced dataset, we choose the T-shirt class as the majority class and the pullover class as the minority class. The majority and minority class samples are 3,000 and 600, respectively, which means the imbalance ratio is 5:1. Considering that the feature size is similar to the number of samples, we set the feature extraction ratio to 0.1. Table 3 shows the result of the experiment.

**Table 3:** Comparisons between imbalance learning algorithm and our proposed method in terms of AUC.

	**SMOTE**	**RandomForest**	**RUSboost**	**Balance bagging**	**LMNN ensemble**	**ImLMNN ensemble**
Fashion-mnist	0.9384 ± 0.0018	0.9523 ± 0.0025	0.9414 ± 0.006	0.9561 ± 0.0043	0.9543 ± 0.0044	**0.9610 ± 0.0012**

*The bold value means the best result among the compared algorithms*.

We can see that our method also has the best performance in the Fashion-mnist dataset. The reason is that our proposed method can deal with the imbalance problem in the image dataset.

### 4.5. The Effect of Parameter

**Figure 4:**
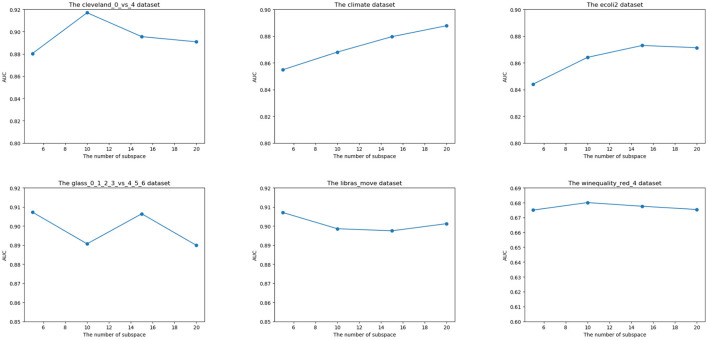
Effect of the number of subspaces on the performance of ensemble on six datasets.

In this section, we show the impact of the parameter in our algorithm. The number of subspaces influences the performance of our ensemble framework. We set the number of subspace to [5,10,15,20]. The parameter experiment result is shown in Figure 4.

For Cleveland_0_vs_4, ecoli2, and winequality_red_4 datasets, the AUC is improved when the value of subspace is increased. However, when the number of subspaces exceeds a specific number, the AUC decreases as the subspace increases. The reason is that the increasing value of subspaces improves the diversity of subspace, while the excessive subspaces introduce redundant information and are harmful to the algorithm's performance. For other datasets, the trend of AUC is diverse due to the uncertainty of the GA algorithm. Considering the algorithm result on the overall dataset, the proposed number of the subspace is 15.

## 5. Conclusion and Future Work

In this article, we propose an ensemble framework to deal with imbalanced datasets. We explore an effective feature space to improve the performance of the subsequent procedure. In addition, we propose an adaptive integrated voting process to assign weights for classifiers. The experiments on various real-world imbalanced datasets, including the imbalanced image dataset, show the superiority of the proposed ensemble framework. Finally, we show the experiment to explore the effect of the parameter.

Future study contains several points: (1) Various methods can transform data into other feature spaces, so choosing appropriate methods should be considered. (2) A more effective adaptive weight process should be explored to assign weight based on the performance of the base classifiers.

## Data Availability Statement

Publicly available datasets were analyzed in this study. This data can be found at: http://archive.ics.uci.edu/ml/index.php.

## Author Contributions

ZL, HW, and KY contributed to conception and design of the study. All authors contributed to manuscript revision, read, and approved the submitted version.

## Funding

The work described in this study was partially funded by the National Key Research and Development Program of China under Grant 2019YFB1703600, in part by the National Natural Science Foundation of China No. 62106224 and Key-Area Research and Development Program of Guangdong Province (No. 2018B010107002).

## Conflict of Interest

The authors declare that the research was conducted in the absence of any commercial or financial relationships that could be construed as a potential conflict of interest.

## Publisher's Note

All claims expressed in this article are solely those of the authors and do not necessarily represent those of their affiliated organizations, or those of the publisher, the editors and the reviewers. Any product that may be evaluated in this article, or claim that may be made by its manufacturer, is not guaranteed or endorsed by the publisher.
